# Between Policy and Risk Communication: Coverage of Air Pollution in Ghanaian Newspapers

**DOI:** 10.3390/ijerph192013246

**Published:** 2022-10-14

**Authors:** Samuel Agyei-Mensah, Elvis Kyere-Gyeabour, Abraham Mwaura, Pierpaolo Mudu

**Affiliations:** 1 Department of Geography and Resource Development, University of Ghana, Legon, Accra P.O. Box LG 59, Ghana; 2 Environment, Climate Change and Health, World Health Organization, 1211 Geneva, Switzerland

**Keywords:** air pollution, health risks, media, public policy, Ghana

## Abstract

Mass media plays an increasingly persuasive role in orienting political decisions, shaping social agendas, influencing individuals’ actions, and interpreting scientific evidence for the public. With growing scientific understanding of the health, social and environmental consequences of air pollution, there is an urgent need to understand how media coverage frames these links, particularly in Low- and Middle-Income Countries. This paper examines how the Ghanaian print and electronic media houses are covering air pollution issues given increased efforts at reducing air pollution within the country. The main goal of this work is to track the progress of policies to reduce air pollution. We used a qualitative content analysis of selected newspapers (both traditional and online) between the periods 2016 and 2021 and we found that articles on air pollution have been increasing, with more reportage on impact and policy issues compared to causes of air pollution. A focus group with six members of the media confirmed an interest in covering health and environmental issues, particularly coverage of specific diseases and human-interest pieces. This increasing attention is likely associated with intensifying local, national, and international action to improve air quality in Ghana, and growing awareness of the health impacts of air pollution.

## 1. Introduction

There has been growing interest in analysing coverage of air pollution in the media globally in recent years [1,2,3,4,5]. Known colloquially as the fourth estate for the powerful role the media plays in shaping social and political reality, increasing attention has been placed on understanding changes in public and political awareness of the impacts of air pollution on public health as a result of media framing [6,7,8]. Such research is particularly crucial in Low- and Middle-Income Countries (LMIC) which experience poor knowledge transfer of the health risks of air pollution [8]. These analyses are partly the result of attempts to measure the impact of international and governmental efforts at reducing air pollution, and track policy and process indicators. For instance, in September 2015 the United Nations General Assembly adopted the Sustainable Development Goals (SDG) with seventeen major themes and air pollution as integral to the success of Goal three (SDG 3), Goal seven (SDG 7) and Goal eleven (SDG 11) (see [9]). SDG target 3.9.1 specifically calls for a sustainable reduction in deaths and illnesses from air pollution.

In addition to the United Nation’s Sustainable Development Goals, many international organizations have paid growing consideration to the issue of air pollution and health. The case of Ghana, and the Greater Accra Metropolitan Area (GAMA) in particular, is of relevance because multiple international, national and local stakeholders have collaborated to assess, report and shape the political agenda on air pollution [10]. International research projects have been carried out focusing on air pollution in Ghana [11]. Over the last few years reports have been produced by UNEP [12], Health Effects Institute [13] and the World Bank [14]. The Climate and Clean Air Coalition (CCAC) has funded several projects, and Ghana was a founding partner, and currently serves as co-chair of the Coalition along with the USA [15]. The World Health Organization (WHO) has promoted Accra as a pilot case study for the Urban Health Initiative (UHI) focusing on household energy, transport, land use and waste since 2016 [16], and has conducted awareness raising activities through the BreatheLife campaign [17]. Other projects are being carried out in collaborations between universities and Ghanaian authorities, including longstanding efforts focused on household air pollution (see for example [18]).

The Accra Metropolitan Assembly has been promoting a policy of greening the city, reducing air pollution and mitigating climate change, with measures taken to address emissions from different sectors, and in 2018 a Greater Accra Metropolitan Area Air Quality Management Plan was launched [19]. The city’s first Resilience Strategy in 2019 [20] and also the Accra Climate Action Plan in 2020 [21], identified health impacts as a key challenge and potential co-benefit of the plans. In 2019, Accra’s engagement efforts with informal waste collectors to reduce emissions and modernize waste management was recognized as one of the world’s seven best climate projects [22]. Additionally, the Ghanaian national government, with international support, discussed and approved new air quality standards and an action plan in 2019 [23].

Within all these policy initiatives and projects, communications strategies were developed to connect with media, policymakers, community leaders, students and other stakeholders, and engage their support for measures to reduce air pollution. By law, Ghana Environmental Protection Agency (EPA) has the overarching responsibility of governing the preservation of air quality and has played a pivotal role in raising awareness and introducing policy measures to address air pollution at the national level. In addition to Ghana EPA, the Accra Metropolitan Assembly (AMA), and Ghana Health Service (GHS) have worked to convene key stakeholders by hosting media workshops, town hall meetings and trainings for clinical and public health workers. These communications and stakeholder engagement efforts have been amplified by the significant increase in the number of scientific and technical publications examining air pollution in Ghana in recent years (see [24,25,26,27,28]).

A qualitative content analysis of popular print and online newspapers was conducted to examine how the mass media are covering air pollution issues in Ghana, with the goal of tracking the progress of these global, national, and local efforts to reduce air pollution. The study draws on analysis of newspaper articles covering the period 2016 to 2021. We analysed the publications through the lens of three news themes: sources of emissions, health impacts and policies concerning air pollution. Text mining and network analysis was conducted to gain further qualitative insights by mapping thematic relationships in the overall news coverage of air pollution.

The main questions were: how have Ghanaian newspapers framed the issue of air pollution and reported on its causes, health impacts and policy measures? Which trends can be identified in the five-year period when policymaking, training, enforcement, or awareness raising activities were carried out to address air pollution? A focus group with 6 key informants from 4 media houses, was also conducted to understand editorial assignments for coverage of environmental health issues, discern media slant on air pollution, and examine research sources for scientific content.

To the best of our knowledge this is the first study to assess media coverage of air pollution issues in Ghana. Previous studies by [29,30] analysed coverage of environmental issues broadly and did not focus on air pollution as well as other media analysis that focus on soil and water degradation [6]. We use the media as a lens here because of its pivotal role in society as a purveyor of scientific evidence and a bridge between experts, decision makers and the public [3].

### Coverage of Air Pollution and Health by Newspapers: An Overview

In this section, we offer an overview of studies on media coverage of air pollution and the communication of associated health risks, with emphasis on newspapers. In the past decade, there has been a significant expansion from consumption of traditional mass media—broadcast television, newspapers, radio—into consumption of new media through the internet and mobile phone communications. However, traditional print and broadcast media remain an important and influential source of information, and often drive the overall knowledge of current events [31].

Several factors affect the coverage of media issues. Theoretically, attempts have been made to situate these factors within three dominant and interrelated conceptual perspectives: agenda setting, priming, and framing [32]. Agenda setting refers to how media emphasis of issues influences public perceptions of their relative importance; priming is the media influence on public adoption of particular issues as measures of political performance; and framing refers to media perspectives constraints, and modes of coverage which influence public understanding and views of issues. Taken together, these conceptual frameworks can allow researchers to gain an overview of the influence mass media has on their audiences’ perception of an issue.

The analysis of newspapers for environmental issues has a long tradition but only recently has media coverage of air pollution become the focus of in-depth investigations [2,4]. Most earlier studies were from high income countries, and it is only recently that studies have focused on middle- and low-income countries [3]. The framing of newspaper stories on air pollution in Delhi between 2011 and 2016 was examined [4]. This followed an episode when the Indian capital was characterized as one of the most polluted cities in the world. The study was based on content analysis of Indian newsprint: Times of India, Hindustan Times, and the Hindu. They found that personal level causal attributions, particularly cars, were mentioned more frequently than societal level or other causes such as industrial emissions and weather. In a similar study on newspaper coverage in Delhi, India, to uncover social perceptions surrounding the debate on air pollution in Delhi, India [33]. They examined how the causes, effects and solutions of this environmental problem are framed by the print media and narrated by non-profit organizations involved in the issue. The findings of the study showed that both the media and non-profit community in Delhi present transportation as one of the leading causes of air pollution, and one of the dominant adverse risks to health. However, the study focused on only the news media in English and did not cover the perspectives of Hindi newspapers.

Coverage of air pollution by the news media has also attracted the attention of Chinese scholars in recent times. The news media in China has been useful for educating the public about the health threats of air pollution [34]. Using 22 articles published in Baidu News, Sun et al. [34] assessed the news coverage of scientific studies concerning outdoor air pollution and its health effects and examined the gap between Chinese scientific research and the media reports. The results of the study showed that national news outlets gave little attention to the scientific findings of Chinese researchers. A recent study examined the impact of air pollution on the media slant of publicly listed firms in China [35]. Using a large panel of air quality and media data at the city level, they found that worse air quality generally leads to a more negative media slant. When the air quality falls from lightly polluted to heavily polluted, the number of negative sentences in a news article increases by about 1%. They also found that the effect of air pollution on media slant is stronger for firms in heavy polluting industries. These results suggest that the level of air pollution affects media slant.

Several studies from high income countries have provided detailed analysis of the relations between media and air pollution issues. In the United Kingdom, a recent study examined controversies and debates on poor air quality along Oxford Street in London [1]. The study analysed 1594 newspaper articles on air pollution that appeared in five British newspapers, The Guardian, Financial Times, The Independent, The Daily Telegraph and The Times, between January 1997 and March 2017. The paper discusses five critical discourse periods amongst others during that time and shows how a number of critical discourse moments have been able to spike media attention and shift the terms of the debate. The paper argues that media processes should not only be understood in cyclic terms, but can also trigger non-linear iterative dynamics, leading to upward spirals characterized by thresholds and a gradually increasing level of interest overall, in this case raising the profile of London’s poor air. In the United States, a study examined how health risks and precautionary measures concerning air pollution are reported using two regional newspapers, the Fresno Bee and the Bakersfield Californian, from the Californian San Joaquin Valley and two national newspapers, the New York Times and the Washington Post [2]. The results of the study showed that newspaper reports on air pollution do not promote raising environmental health literacy as the newspapers have the tendency of failing to report on the impacts air pollution can have on health. Another study on media coverage of air pollution in the United States by Mayer [36] used twenty years of newspaper articles from three newspaper sources (New York Times, Los Angeles Times, and the Washington Post), to explore the print media’s coverage of the relationship between asthma and air pollution. The study found little consistency in the coverage of whether asthma is caused directly by air pollution or exacerbated by exposure. Looking at the possible range of information given on the effects air pollution caused by bushfire smoke has on human health and climate change, Linnenluecke and Marrone [37] examined 512 Australian newspaper articles published over a 5-year period, 2016–2021, that report on air pollution. They noted that a temporary surge in articles occurs during the unusually severe 2019/2020 Black Summer bushfires. However, most articles are limited to general statements about the health impacts of bushfire smoke with only 9 percent of the articles in the sample mentioning an explicit link between bushfire smoke inhalation and cardiovascular and respiratory problems or increases in mortality risk.

In Africa, the only paper we found on media coverage of air pollution was a study by John [38] in Nigeria. The study examined how Nigerian newspapers covered air pollution to raise awareness and prompt the government’s attention to it. Analysing one-year daily editions of three national newspapers (The Guardian, Daily Sun, and Vanguard) from July 2016 to June 2017, results showed poor reporting of air pollution by the newspapers. The study [38] concluded that overall, the level, placement and style of the news reports were not enough to create public awareness, stir up public discourse and cause the government to act concerning air pollution in the country. Even though there are limited studies on media coverage of air pollution in Africa, there are comparatively more studies on media coverage of environmental issues such as climate change (see [39]).

Additionally, some studies in Ghana have looked at media framing of environmental issues more broadly, not focusing on air pollution (see [29,30,40]). It is evident from the above overview that not much scholarly attention has been devoted to studies on newspaper coverage of air pollution in Africa. Overall, the results of the papers indicate: (1) coverage of single visible causes, for example transport, not necessarily the main source of air pollution, was mentioned more frequently than multiple causes and activities; (2) adverse health effects were usually ignored or mentioned briefly, and newspapers did not promote raising environmental health literacy; (3) attention to results of research publications was low; (4) the level of air pollution affects media slant; (5) political or scientific frames were applied more frequently in news coverage compared to health impacts, even in policy discussions; (6) media coverage occurred both in cyclic terms, and also in non-linear iterative dynamics, leading to upward spirals of gradually increasing levels of overall interest; (7) temporary surges in articles occurred during unusually severe pollution episodes.

## 2. Materials and Methods

The study was based on content analysis of selected media houses in Ghana covering the period from the first of January 2016 to 31 December 2021 as well as focus group discussions of a select media houses. Ghana has a thriving newspaper business with circulation growing each year. Private print media was almost absent from the Ghanaian media landscape until 1992, when the current constitution came into being. We focused on the following media houses: Daily Graphic; Daily Guide; Ghanaian Times; The Chronicle; The Herald; The Mirror; MyJoy Online; PeaceFM Online; and Citi Newsroom Online (Table 1). Daily Graphic has an online version of the print called Daily Graphic Online. To avoid duplication, we did not include articles featured on the Daily Graphic online.

The Daily Graphic, now a state-owned daily newspaper, was introduced on the news stand on 2 October 1950, as one of a chain of newspapers owned by private interest, the Daily Mirror Group of London. It was sold to the Government of Ghana which eventually took over in 1965. According to Dzisah [41], The Daily Graphic has the largest nationwide readership and leads the newspaper industry with a daily circulation of over one hundred thousand (100,000) copies. The Daily Guide is published by Western Publications Limited. It is a privately-owned daily newspaper. It is published in Accra, and it comes out six (6) times per week and is regarded as the most circulated privately-owned newspaper in Ghana with a circulation of about twenty-two thousand copies a day [41]. GeoPoll also measured average newspaper readership each day for some of the Ghanaian newspapers. GeoPoll found that Daily Graphic was the most popular daily newspaper, at 1.5 million readers per day, followed by the Daily Guide at 726,000, and the Ghanaian Times in third at 532,000 [42].

In selecting the newspapers, we considered the assertion by [43] to focus on ‘leading’ newspapers which are characterized by in-depth and high-quality media coverage, and which tend to set the terms of the debate, including driving coverage of issues by other newspapers. We examined both print and the electronic media (see Table 1).

Print media newspapers were obtained from archives of the University of Ghana Balme Library. For the electronic media houses, the online archives of the selected online news sources were used for the identification of air pollution publications. The following information was extracted from each article that reported on air pollution: headline of the article; publication type (i.e., electronic or print); theme of the article (i.e., cause, impact, or policy); and publication date. The KH Coder text mining software created by Koichi Higuchi at Ritsumeikan University, Kyoto, Japan [44] was used to analyse the extracted air pollution publications. Employing text mining to analyse qualitative data has been used in different research including works of [45,46]. A word cloud, a visual representation of the frequency of words in qualitative data, is used below to show the most frequently occurring words in a dataset.

A semantic network is a logic-graph constructed from a set of vertices and nodes to show the relationships which exist within and between qualitative datasets [47,48]. The qualitative content analysis of the extracted data included charts, tables, word frequency, word cloud and semantic network (co-occurrence) analysis.

A decision was also taken to use a focus group to analyse some of the results of the content analysis with more details [49]. A focus group with 6 select media representatives from 4 media houses was organized to explore their views, priorities and perspectives of news coverage of air pollution and health issues in Ghanaian media. Two representatives each from the Daily Graphic and MyJoy Online, and one each from PeaceFM and MetroTV attended the focus group discussion. The focus group interview followed a semi-structured process, with some preselected probing questions and flexible follow-up questions to understand editorial decisions, specific messages covered, audiences targeted, additional platforms used, and media slant related to specific target groups. The duration of the focus group was 1 h, with notes, as well as a digital recording, taken by the moderator. Questions included: (1) In your media company, how are assignments made for coverage of air pollution and environmental health issues? (2) In your media company, is a particular person assigned to cover stories on air pollution or environmental health? (3) Has your knowledge on air pollution increased during the period 2016–2021? (4) What are your sources of air pollution and health related data? The analysis of the information was performed without the use of a specific software but using the notes and the transcript of the conversation organized by major themes, separated in sections.

## 3. Results

**Figure 1:**
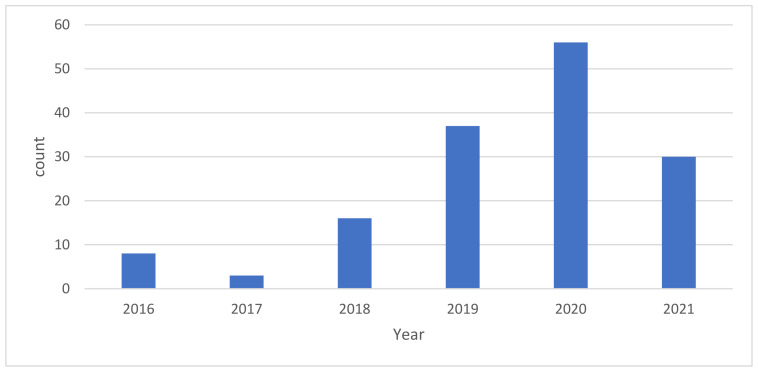
Air pollution publications per year from 2016–2021.

We identified 150 articles between the period 2016 and 2021 from the total pool of all published articles. Articles on air pollution have been increasing over the years with some dips in 2017 and 2021 (Figure 1). The highest number of reported articles of 56 was in 2020. The lowest number of reported articles of 3 on air pollution was recorded in 2017.

**Figure 2:**
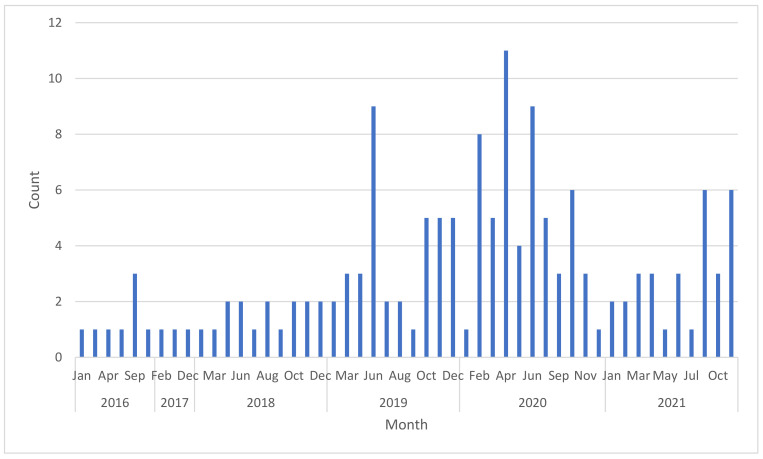
Air pollution publications per month from 2016–2021.

There is generally no major trend regarding the month of publication of air pollution related news (Figure 2). However, it can be observed that most air pollution publications were during the Harmattan season, during which dust particles are transported across the continent from the Sahara Desert to the Atlantic Ocean. These seasonal changes are accompanied by major changes in air quality levels. The air pollution publications were not, however, influenced by these seasonal environmental changes. Overall air pollution publications saw a steady rise month on month after 2017. The highest number of air pollution publications of 11 was recorded in April 2020 (Figure 2).

**Figure 3:**
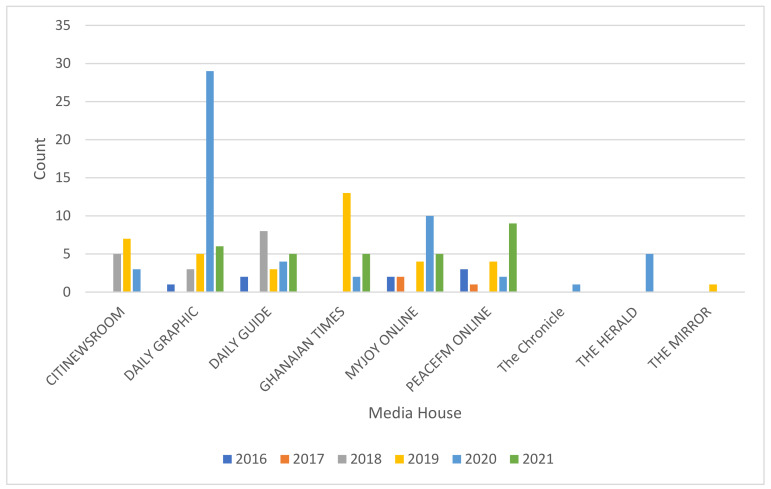
Air pollution publications by Media house per year from 2016–2021.

The traditional print media houses such as Daily Graphic, Daily Guide and the Ghanaian Times recorded the highest number of articles (Figure 3). Interestingly, the electronic media houses such as Myjoyonline and Peacefm, established more recently in the 1990s, were also reporting more air pollution articles. The Daily Graphic, a traditional print media, reported the highest percentage of 29% of air pollution articles during the period of 2016 to 2021. Myjoyonline, an electronic media reported 15% of air pollution publications, as did Daily Guide, a traditional print media. The lowest percentage of 1% was recorded by two traditional media houses, The Chronicle and The Mirror, for air pollution publication during the period of 2016 to 2021.

**Figure 4:**
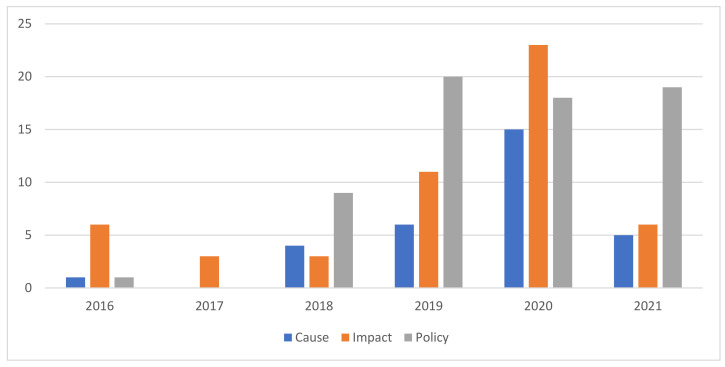
Air pollution publications by Theme per year from 2016–2021.

If we consider air pollution publications by thematic focus, we can distinguish three main categories: causes, policy and impact (Figure 4). The causes category focuses on publications that highlight activity or occurrences that bring about air pollution. The policy category focuses on publications that include policy, policy initiatives, and policy driven events about air pollution. The impact category focuses on publications that highlight the resulting effects and consequences of air pollution on the environment and human health. This includes both long-term and short-term effects from air pollution. The air pollution publications were largely focused on policy, which accounted for 45% of air pollution publications from 2016 to 2021 (Figure 4). The highest thematic reporting over the study period was the impact category in 2020.

**Figure 5:**
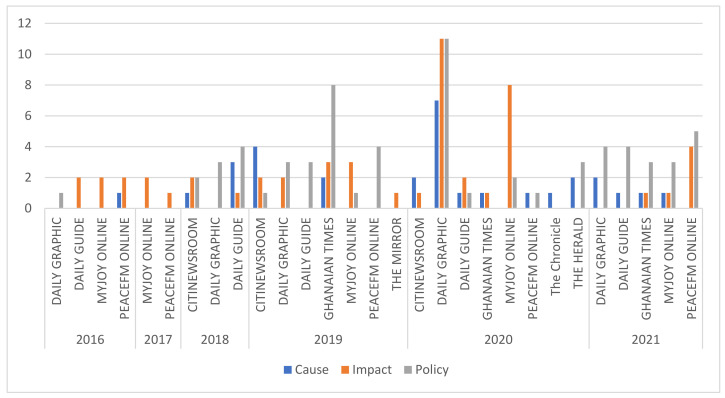
Air pollution publications by Theme per Media House from 2016–2021.

The focus of the thematic air pollution publications indicates that reporting on all three themes was done by Citinewsroom in 2018, 2019, Ghanaian Times in 2019, 2021, Daily Graphic in 2020, Daily Guide in 2020 and Myjoyonline in 2021 (Figure 5).

**Figure 6:**
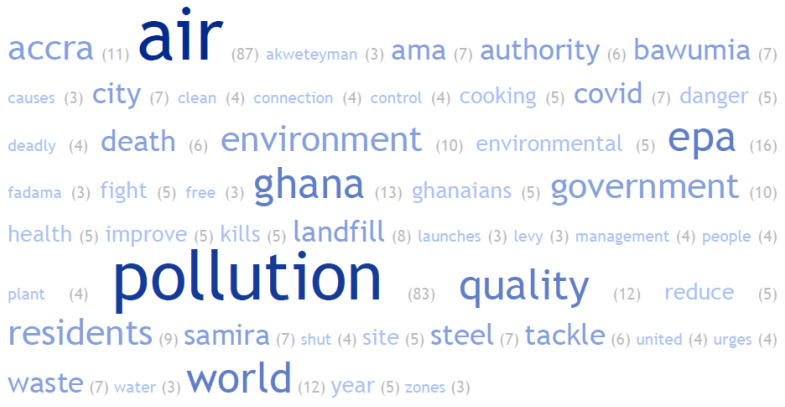
Word cloud of Air Pollution publication headlines.

Analysis of the headlines that captured the media stories offers additional insights. With respect to causes of air pollution, issues such as burning of waste, cooking, motorcycles, and taxis (traffic induced air pollution), landfill sites and galamsey (small-scale, informal gold mining and refining) featured in some of the headlines (see Table A1 in Appendix A). A total of 529 words were extracted from the headlines and the most frequently occurring 100 words were used to generate the word cloud (Figure 6). The highest occurring words were “EPA”, “Ghana” “quality” and “world”.

**Figure 7:**
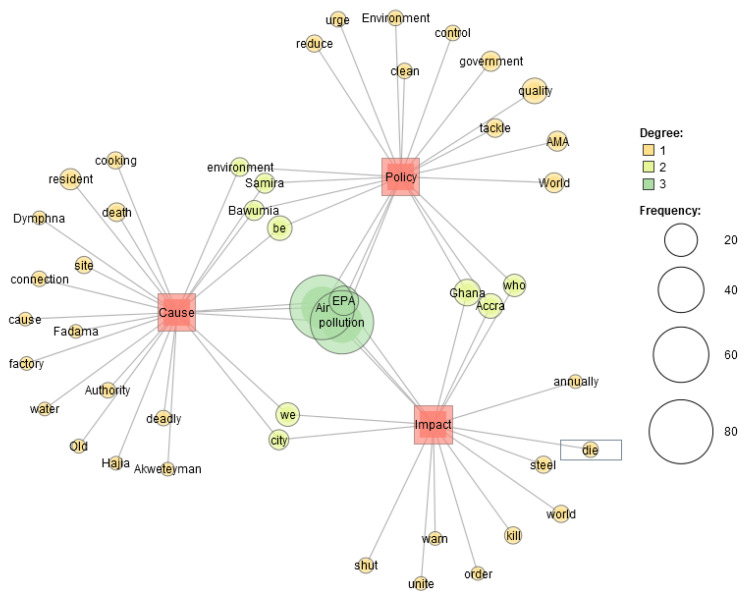
Semantic Network of air pollution publications by thematic focus.

**Figure 8:**
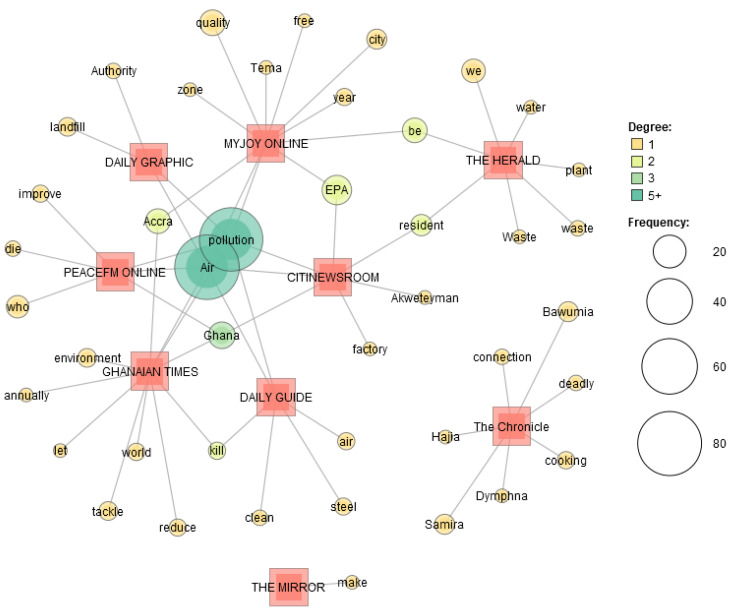
Semantic Network of air pollution publications by media house.

The semantic network analysis for air pollution publications by thematic focus (‘cause’, ‘impact’ and ‘policy’) (Figure 7) found that ‘cause’ was found to have the most connections. Connecting words between the different thematic areas were identified in the degree 2, one of these connecting words was ‘Samira’ and ‘Bawumia’ which hits of the attention media reportage give to personalized air pollution reports. The name of the second-lady Samira Bawumia recorded 14 co-occurrences more than ‘environment’, ‘health’ and ‘death’ which all recorded lower co-occurrence in the semantic analysis. The semantic network analysis for air pollution publications by media house (Figure 8) identified media houses with similar headline reportage on air pollution for the period under study. These media houses were the most popular media houses in Ghana. However, media houses like Chronicle, Mirror and Herald were identified to have headline reportage that was not like the headlines of other popular media houses. There were 5 major networks from the dataset with similar headline reportage connecting at least two media houses.

As indicated earlier, we also conducted focus group discussions with six media personnel from four media houses. Of the four outlets, only the Daily Graphic could be considered a structured newsroom having a dedicated Health and Environment desk, with three reporters and an editor being automatically assigned environmental health-related stories. Interestingly, the newspaper started with a health desk, and over time the reportage often made links to the environment and led to a decision to form a joint health and environment desk. The other outlets assigned air pollution or environmental health stories somewhat randomly, depending on reporters’ availabilities, or which political figure or agency was involved. While it may test the readers credulity, two reporters claimed that even the most technical air pollution and health stories would generally be assigned to the lifestyle or human-interest reporters, who then shape the focus and the background research of a given story based on their own interests or experience. In all cases, the reporters were expected to be generalists, able to take assignments of any topic. Apart from the Daily Graphic, the reporters did not feel that their knowledge of air pollution and health issues had improved over time, though they mentioned participating in a capacity building workshop on e-waste hosted by Germany and the government of Ghana.

Responses from the key media informants interviewed during the focus group confirmed an effort at agenda setting and priming, claiming “there are two audiences for behaviour change messages: the general public and the policy-makers,” according to one editor present. One reporter felt that a story would still be incomplete if it did not tell the audience “what to do about an issue”. There was also an acknowledgement of a reciprocal dynamic with the media following the interests of the public when making decisions about news coverage and following the priorities of policy-makers. “Once stakeholders are engaged and pushing for an issue, the journalists will pick it up,” said one reporter. Primary sources of data for researching stories were varied, depending on the geographic scale of coverage and the angle of the story chosen by the reporter. Only representatives from the Daily Graphic felt that their knowledge of air pollution and health issues had improved over the period, with the other outlets citing random assignment of the topic as an impediment to increasing their knowledge of the issue. The reporters cited capacity building trainings hosted by government agencies and NGOs as important opportunities to increase their technical knowledge—including on galamsey mining and e-waste recycling, both major sources of pollution. The media representatives identified science reporting as a key weakness, claiming that experts are often not able to break down technical content, and only a few cases where it was presented with a clear target audience. Columns, opinion pieces and features written by outside experts were welcomed but were often not written in a relatable way. While acknowledging her responsibility as a journalist, one reporter claimed that if she could not understand the technical content by the end of a press conference then she would not file a story—a stunning rebuke to science communicators. Impacts on vulnerable groups including women and children, and street vendors were offered as examples of human-interest angles which could be used to shape air pollution stories. Stories about ongoing sources or air pollution like galamsey mining, or road dust could be of interest to their audiences. Moreover, stories about specific diseases were considered good hooks to tell air pollution human-interest stories. Notably, one outlet was asked by readers to break down non-communicable diseases one at a time, following a story on their increasing incidence. Twi and local language radio stations were also raised as possible channels for air pollution and health reportage.

## 4. Discussion

This study sought to examine media coverage of air pollution causes, health impacts and policy solutions in Ghana covering the period 2016 to 2021. The study provides the first assessment of popular media coverage of air pollution issues and identified many developments in media reporting on air pollution. Our findings indicate that the media has created public attention toward air pollution and that coverage of air pollution issues have increased since 2016 coinciding with increased attention to air pollution in Ghana by local, national, and international agencies including the WHO during the period, including increased outreach to the media. The study also finds that causes of air pollution have been underrepresented in coverage compared with policy and impact.

The monthly reports of air pollution coverage indicate that reporting is frequently done from October to March of each year during the Harmattan season, a period during which dust particles are transported across the continent from the Sahara Desert to the Atlantic Ocean. On such occasions, elevated levels of air pollution are experienced [28]. While some of the causes of air pollution such as burning of tires, traffic induced air pollution, landfill sites, illegal mining (galamsey) and burning of waste featured in the headlines, neither the varied illnesses resulting from air pollution nor the populations that are particularly vulnerable were specifically mentioned in the reportage. For instance, none of the banner headlines touched on the health effects of air pollution such as cardiovascular diseases, cancers, and respiratory diseases. As is known, the effects of air pollution on cardiovascular risks may be felt more slowly, but evidence of the health risks received no headline coverage. There is therefore the need for more training about the impacts of air pollution on human health amongst media professionals.

With regards to the major drivers of media coverage on air pollution, we observed that the activities of the Ghana EPA as well as the activities of international organizations have effectively raised awareness and visibility about air pollution issues. For example, some of the banner headlines on policy attested to the partnership between international organizations such as the WHO and the Ghana EPA in drawing attention to and addressing air pollution. The semantic network analysis of the air pollution publications by thematic focus converged on highlighting the role of the Ghana EPA, whose activities had an important influence in addressing air pollution in the country, and in driving key messages. Another convergence in media coverage came from headlines based on policy statements of public officials such as Ministers of State, Municipal Chief Executives, Diplomats, and the spouse of the Vice President. There were also yearly events influencing coverage such as the celebration of the International Day of Clean Air for blue skies, as well as World Environment Day. Additionally, the COVID-19 pandemic has influenced air pollution coverage as evidenced in some of the banner headlines since the onset of the pandemic. The general perception is that the lockdown periods may have improved the quality of air pollution because of reduced traffic on the roads (see also [50]). Despite the visibility given to public officials, yearly events, and international and national bodies in the coverage of air pollution issues, we did not see headlines covering the work of the academic and scientific community (see also [2]). Academics should endeavour to communicate the growing scientific evidence through newspaper articles and other mass media channels, designing messages which reach stakeholders across relevant sectors and disciplines, as has been seen in recent newspaper coverage of noise pollution research in Accra [51]. Researchers can play a key role in providing evidence to strengthen reportage on health impacts and fill the gaps in communicating sources of air pollution.

While the content analysis did not find evidence of an automatic or manifest agenda setting process linking media coverage of air pollution and health to political or public perceptions of the issue, framing around the issue indicated a sense of urgency with words like “kill” or “deadly” associated with both impacts and sources of air pollution. In one case, a single outlet dominated the overall coverage of a particularly egregious polluter in the municipality of Akweteyman, following the story over a period starting with community complaints and finally with the Ghana EPA shutting down the site after a series of citations. Storytelling as ongoing coverage of this particular case can be considered a form of agenda setting as it shifted the relative importance of air pollution for the readership, created a sense of familiarity with the issue and broke down the complexities of taking action by drawing it out in real-time. However, this is a key example of coverage of a single source of pollution, with little joint coverage of the adverse health impacts. Headlines also indicated some effort at media priming, with editorial choices to promote government or public action on air pollution, and laudatory editorials about the actions of particular officials on the issue. There are latent indications that media coverage of official air pollution actions and messages drove public understanding of the issue in Ghana during the period. This effort at agenda setting and priming was further confirmed by key media informants in the focus group.

## 5. Conclusions

Despite the novelty of this study, it suffers from some limitations. To begin with, while the traditional print media has long been central to informing the public and focusing public attention on critical environmental issues [29,30] social media may also be playing an important role in covering air pollution issues. Research in the US suggests that amongst younger age groups, internet sources are preferred to traditional media as sources of information [52]. Journalists in the focus group cited interactions with their audiences on social media as driving coverage of an issue. Future studies should include social media since the youth are more attentive to this medium of communication. Additionally, broadcast media can also provide valuable sources of information, particularly radio programming in local languages, and can also play a significant role in influencing public perceptions about air pollution, even though most of the broadcast media depend on the traditional print media sources for information [53]. Conducting additional interviews of media professionals and key advocates for clean air could shed more light on how air pollution issues are perceived by media professionals and the political, economic, and other interests that affect framing and agenda setting [43,54]. Coverage of air pollution issues may also be affected by the journalist’s background and training as not all journalists may be conversant with issues on air pollution, climate change and public health. Analysis of the coverage spotlighted sometimes conflicting coverage regarding the number of deaths from air pollution in Ghana, a further indication of the need for deeper engagement between scientists and journalists. Interviewees in the focus group indicated a need to increase background knowledge and capacities around environmental health in the media.

In conclusion, the media is playing an important role in raising awareness about air pollution in Ghana. However, more attention should be focused on giving visibility to the causes and health effects of air pollution.

## Figures and Tables

**Table 1:** Selected Media Houses in Ghana.

Media House	Established	Published	Ownership	Readership *
Citinewsroom	2004	Daily	Private	84,000 per day
Daily Graphic	1950	Six Days	Public	1,519,000 per day
Daily Guide	1984	Six Days	Private	726,000 per day
Ghanaian Times	1957	Six Days	Public	530,000 per day
Myjoyonline	1995	Daily	Private	83,000 per day
Peacefm Online	2000	Daily	Private	111,000 per day
The Chronicle	1990	Six Days	Private	1,730,000 per day
The Herald	1996	Six Days	Private	
The Mirror	1953	Saturdays	Public	416,000 per day

* source: [31].

## Data Availability

The data presented in this study are available here. https://doi.org/10.17894/ucph.4de52249-41e3-4908-b497-fc7df3effa6c.

## Appendix A

**Table A1:** Summary of headlines on air pollution by media houses in Ghana for 2016–2021.

Date	Media House	Headline	Theme
7 January 2016	MYJOY ONLINE	Air pollution kills 6.5 million people every year	Impact
13 February 2016	PEACEFM ONLINE	Polluted air causes 5.5 million deaths a year. New research says	Cause
22 April 2016	MYJOY ONLINE	6500 Ghanaians die every year from air pollution	Impact
14 May 2016	PEACEFM ONLINE	Air pollution rising in the world’s poorest cities.	Impact
27 September 2016	DAILY GRAPHIC	Airlines embrace US $24 Billion pollution plan	Policy
27 September 2016	PEACEFM ONLINE	17,524 Ghanaians died from air pollution—Code for Ghana	Impact
28 September 2016	DAILY GUIDE	92% Air polluted—WHO	Impact
13 December 2016	DAILY GUIDE	Pollution kills 12.6 million	Impact
16 February 2017	MYJOY ONLINE	Air pollution emergency in Nigerian city	Impact
12 June 2017	MYJOY ONLINE	Air pollution causes brain damage in babies—UNICEF	Impact
28 December 2017	PEACEFM ONLINE	17,000 die annually from air pollution in Ghana	Impact
11 February 2018	DAILY GRAPHIC	We need concerted efforts in tackling air pollution—Mrs. Bawumia	Policy
19 March 2018	CITINEWSROOM	La residents to sue EPA, EDISAW over ‘toxic’ rubber factory	Cause
6 April 2018	DAILY GUIDE	Green Republic project to plant 10, 000 trees in Tamale	Policy
26 April 2018	DAILY GUIDE	Cleric Bemoans Environmental pollution	Impact
6 June 2018	CITINEWSROOM	“Ghana needs holistic ways to fight environmental degradation”—Prof. Mike Oquaye	Policy
29 June 2018	DAILY GUIDE	DCE champions tree planting	Policy
9 July 2018	CITINEWSROOM	British High Commissioner complains about ‘disgraceful’ air pollution near his home	Impact
16 August 2018	CITINEWSROOM	AMA launches air quality management program	Policy
17 August 2018	DAILY GUIDE	AMA Launches Breathelife campaign	Policy
23 September 2018	CITINEWSROOM	More people may die from air pollution in Ghana—WHO warns	Impact
19 October 2018	DAILY GRAPHIC	Abibiman metal insert—Device to reduce household air pollution	Policy
31 October 2018	DAILY GRAPHIC	Design local solutions for air pollution—Samira Bawumia to WHO	Policy
9 November 2018	DAILY GUIDE	Air pollution: Authorities’ Failure	Cause
9 November 2018	DAILY GUIDE	British envoy condemns Tyre burning near his residence	Cause
9 December 2018	DAILY GUIDE	Air pollution killing more Ghanaians	Cause
20 December 2018	DAILY GUIDE	Healthy air: Healthy people	Policy
1 October 2019	GHANAIAN TIMES	Kassena-Nankana rolls out 5 years plan on clean cooking	Policy
13 February 2019	CITINEWSROOM	Committee set to investigate pollution by factory at Akweteyman	Cause
14 February 2019	CITINEWSROOM	Akweteyman: EPA orders Toffee Company to cease operation over air pollution.	Impact
13 March 2019	CITINEWSROOM	MD Estate residents complains of air pollution from oil refinery	Cause
13 March 2019	CITINEWSROOM	Royal sweets company given six month to relocate	Impact
22 March 2019	GHANAIAN TIMES	Government planning to introduce electric buses to reduce pollution- Titus Glover.	Policy
17 May 2019	GHANAIAN TIMES	Air pollution kills 20, 000 annually- EPA	Impact
19 May 2019	DAILY GRAPHIC	W.H.O warns of air pollution danger	Impact
24 May 2019	PEACEFM ONLINE	Adopt cost-effective interventions to address air pollution- Samira Bawumia to WHO.	Policy
6 June 2019	PEACEFM ONLINE	Samira Bawumia calls on Ghanaians to make the environment Safe.	Policy
10 June 2019	MYJOY ONLINE	Alarm on air pollution-who to our rescue?	Impact
18 June 2019	GHANAIAN TIMES	Let’s fight air pollution together	Policy
21 June 2019	GHANAIAN TIMES	Health alert: World Health Organization warns: Ghana’s air pollution dangerous! Calls for action to improve air quality	Impact
21 June 2019	GHANAIAN TIMES	Time to tackle air pollution in Ghana	Policy
22 June 2019	DAILY GUIDE	Declining air quality: Time to act	Policy
27 June 2019	PEACEFM ONLINE	Fighting air pollution: A complex but unavoidable task.	Policy
28 June 2019	PEACEFM ONLINE	Enforcing existing laws could help improve Air pollution- EPA.	Policy
29 June 2019	DAILY GRAPHIC	AWEP educates pupil of Dawhenya Methodist B. Basic School on environmental pollution	Policy
6 July 2019	DAILY GRAPHIC	Ensuring clean air is shared responsibility- public told on World Environment Day	Policy
6 July 2019	GHANAIAN TIMES	Ghana marks world environment day	Policy
6 August 2019	DAILY GRAPHIC	Alarm on air pollution who to our rescue?	Impact
22 August 2019	GHANAIAN TIMES	Air pollution is a major killer in Ghana-EPA	Impact
10 September 2019	THE MIRROR	Air pollution can make you bald	Impact
12 October 2019	GHANAIAN TIMES	Auto sprayers must acquire environment permit	Cause
12 October 2019	GHANAIAN TIMES	EPA to provide data on air quality in Accra	Policy
17 October 2019	DAILY GUIDE	Zoomlion salutes AMA boss	Policy
22 October 2019	CITINEWSROOM	East Legon: EPA probes air pollution allegedly caused by Akwaaba oil refinery	Cause
6 November 2019	GHANAIAN TIMES	Let’s tackle air pollution now	Policy
12 November 2019	DAILY GRAPHIC	EPA set to strengthen air quality control	Policy
12 November 2019	DAILY GUIDE	EPA to begin daily air quality broadcast	Policy
12 November 2019	GHANAIAN TIMES	Reducing air pollution in Accra	Policy
27 November 2019	MYJOY ONLINE	EPA order united steel to shut down over air pollution.	Impact
2 December 2019	CITINEWSROOM	We’re inhaling death from toffee factory fumes—Akweteyman residents	Cause
10 December 2019	GHANAIAN TIMES	Auto sprayers must acquire environment permit	Cause
12 December 2019	MYJOY ONLINE	Journalists urged to champion campaign against air pollution	Policy
26 December 2019	CITINEWSROOM	Air pollution: Madina Municipal Assembly to ensure Nkulenu Complies with safety standards	Policy
28 December 2019	MYJOY ONLINE	WHO statistics shows that Accra’s air highly contaminated.	Impact
17 January 2020	MYJOY ONLINE	Photo of the week: When air pollution becomes nonnal.	Impact
4 February 2020	MYJOY ONLINE	EPA shuts production plant of united steel over air pollution	Impact
5 February 2020	DAILY GRAPHIC	Booming sand business along Elmina beach	Impact
7 February 2020	DAILY GRAPHIC	Mudslide disaster looms at Weija-Ghana Highway Authority	Cause
19 February 2020	THE HERALD	The only commodity we don’t import is water, Galamsey will get us there very soon	Cause
21 February 2020	PEACEFM ONLINE	James Town under research for Air pollution.	Policy
22 February 2020	GHANAIAN TIMES	Accra Air pollution high above acceptable world Standards—U.S. expert	Impact
26 February 2020	MYJOY ONLINE	Of 30 cities with world’s worst Air pollution 21 are in 1 country	Impact
28 February 2020	DAILY GRAPHIC	Avert this environmental time bomb	Impact
9 March 2020	MYJOY ONLINE	MND metals loses $800,000 in 2 years over Air pollution in Tema free zones	Impact
16 March 2020	DAILY GRAPHIC	Kpone landfill site poses danger to residents	Cause
16 March 2020	DAILY GRAPHIC	Upon landfill site poses danger to residents.	Impact
20 March 2020	MYJOY ONLINE	EPA directive against Air pollution at Tema free zones fail	Policy
31 March 2020	DAILY GRAPHIC	Source Sorting: promoting sustainable collection	Policy
1 April 2020	CITINEWSROOM	Air pollution at Lartebiokorshie: Activities at Old Fadama Landfill site endangers health of residents	Cause
5 April 2020	DAILY GRAPHIC	Waste companies appeal to government over landfills closure	Impact
18 April 2020	DAILY GRAPHIC	Why government must ban rubbish burning now. An applause to a minister	Policy
19 April 2020	DAILY GUIDE	Hajia Samira Bawumia and Dymphna Van der “COVID-19, Air pollution and cooking: A deadly connection”	Cause
20 April 2020	MYJOY ONLINE	People exposed to pollution risk COVID-19 infections.	Impact
22 April 2020	DAILY GRAPHIC	Hajia Samira Bawurnia & Dymphna Vander Lans “COVID-19, air pollution and cooking: a deadly connection”	Impact
22 April 2020	DAILY GRAPHIC	COVID-19, air pollution and cooking: a deadly connection	Impact
24 April 2020	DAILY GRAPHIC	How coronavirus has made Ghana’s invisible silent killer of air pollution visible	Policy
25 April 2020	MYJOY ONLINE	Indiscriminate smoke pollution leaves us exposed to COVlD-19- Steel workers at Tema free zone laments.	Impact
27 April 2020	The Chronicle	Hajia Samira Bawumia and Dymphna Van der “COVTD-1 9 Air pollution and cooking: A deadly connection	Cause
28 April 2020	DAILY GRAPHIC	3 landfill sites shut over nonpayment of services	Impact
2 May 2020	DAILY GRAPHIC	Booming sand business along Elmina beach	Cause
11 May 2020	DAILY GRAPHIC	Prioritize waste management in COVID-19 fight—Environment service providers urges government	Policy
18 May 2020	DAILY GRAPHIC	Old Fadama demolition, stubborn slum dwellers or wobby city Authority?	Cause
3 June 2020	DAILY GRAPHIC	2nd Phase of Swiss-funded waste management project launched	Impact
3 June 2020	DAILY GRAPHIC	EPA orders riders steel to shut down over air pollution	Impact
4 June 2020	DAILY GUIDE	Kpone Asokore Landfills to be turned into recreational Centre	Policy
5 June 2020	DAILY GRAPHIC	World environment day marked	Policy
15 June 2020	DAILY GRAPHIC	Waste management agencies donate towards World Environment Day	Policy
23 June 2020	MYJOY ONLINE	Air pollution over Accra and Kumasi improves during COVID-19 lockdown	Impact
24 June 2020	DAILY GRAPHIC	Provide roadmap on pollution control	Policy
24 June 2020	DAILY GUIDE	Frimpong-Baoteng threatens to close down rider steel over pollution	Impact
25 June 2020	DAILY GRAPHIC	Oti landfill finally ready for upgrade	Policy
1 July 2020	DAILY GUIDE	MESTI stops operations of united steel over pollution	Impact
2 July 2020	DAILY GRAPHIC	Mudslide disaster looms at Weija-Ghana Highway Authority	Impact
13 July 2020	DAILY GRAPHIC	How natural leaders influence good environmental practice	Policy
18 July 2020	DAILY GRAPHIC	Re-engineering of Oti landfill site begins.	Policy
22 July 2020	DAILY GRAPHIC	Residents breathe death in Galilee	Impact
7 September 2020	DAILY GRAPHIC	West Mamprusi tackles sanitation	Cause
17 September 2020	MYJOY ONLINE	Government is committed to reducing air pollution-related deaths—Environment minister.	Policy
18 September 2020	CITINEWSROOM	Over 400 United Steel workers laid off after shutdown over air pollution	Impact
12 October 2020	THE HERALD	The only commodity we don’ t import is water, Galamsey will get us there very soon	Cause
12 October 2020	THE HERALD	Upper West residents jubilates-As prez. Cuts Sod for solid waste plant at Kperisi	Policy
23 October 2020	DAILY GRAPHIC	Put used water sachets in pockets, bags—U/E Minister Cautions against littering	Cause
27 October 2020	PEACEFM ONLINE	Motorcycles, Taxi are a source of Air pollution-Muntaka Chasant	Cause
30 October 2020	GHANAIAN TIMES	Ghana wildlife society calls for appropriate disposal of PPE to curb air pollution	Cause
30 October 2020	THE HERALD	Procurement & Feasibility concerns emerge on Zoomlion’s 10 regional Waste treatment contracts	Policy
2 November 2020	DAILY GRAPHIC	Plastic- infused blocks cut building cost, save environment	Policy
7 November 2020	CITINEWSROOM	Nick Solomon writes: Ghana’s poor air quality not safe amidst COVID-19	Cause
25 November 2020	THE HERALD	NGO partners with La Nkwantanang-Madina Assembly to clear choked gutters	Policy
29 December 2020	MYJOY ONLINE	Air pollution a factor in girl’s death, inquest find	Impact
7 January 2021	GHANAIAN TIMES	The Ozone layer, Greenhouse effect and the Quran	Policy
29 January 2021	PEACEFM ONLINE	AMA opens dialogue on air quality promotion	Policy
9 February 2021	PEACEFM ONLINE	AMA Boss lauds Urban Health Initiative to improve air quality in Accra	Policy
28 February 2021	MYJOY ONLINE	AMA expresses commitment to improving air quality in Accra.	Policy
13 March 2021	PEACEFM ONLINE	Government proposes 10 pesewas sanitation and pollution levy	Policy
16 March 2021	DAILY GUIDE	CONIWAS backs “Borla levy”14th March, 2021. Government introduction of 10 pesewas Borla tax for a clean Ghana. The best way to go	Policy
27 March 2021	DAILY GRAPHIC	Sanitation, pollution levy must be welcoming news but…	Policy
19 April 2021	PEACEFM ONLINE	Hawkers, motorists in danger from air pollution	Impact
24 April 2021	GHANAIAN TIMES	Air pollution kills 1000 Ghanaians annually	Impact
29 April 2021	MYJOY ONLINE	Let’s desist from burning waste to reduce air pollution—AMA	Cause
7 May 2021	GHANAIAN TIMES	Lets prepare to fight Greenhouse emissions with nuclear energy	Policy
25 June 2021	DAILY GRAPHIC	WHO, University of Ghana tackle air pollution	Policy
29 July 2021	DAILY GUIDE	Leverkusen Chemical blast likely released toxins into air	Cause
6 November 2021	DAILY GRAPHIC	Urban Health initiative in Accra reviewed.	Policy
11 November 2021	DAILY GRAPHIC	Stop air pollution	Cause
28 November 2021	DAILY GRAPHIC	Include air pollution control measures in policy making decisions—World Bank urges government.	Policy
8 September 2021	DAILY GRAPHIC	Air pollution in Accra still high—EPA.	Cause
22 October 2021	GHANAIAN TIMES	Injunction against Empire cement struck out.	Cause
12 November 2021	GHANAIAN TIMES	Commit more resource into clear energy solutions -Samira Bawumia	Policy
9 November 2021	DAILY GUIDE	World Bank reps engage editors on air pollution	Policy
11 September 2021	DAILY GUIDE	Air pollution: Authorities’ failure	Policy
8 September 2021	DAILY GUIDE	EPA marks clean air for Blue Skies Day.	Policy
28 September 2021	PEACEFM ONLINE	Include air pollution control measures in policy decisions—World Bank urges Government.	Policy
8 September 2021	PEACEFM ONLINE	Air pollution in Accra still high—EPA	Impact
23 September 2021	PEACEFM ONLINE	Air pollution: Even worse than we thought -WHO	Impact
25 June 2021	PEACEFM ONLINE	WHO University of Ghana tackles air pollution.	Policy
18 October 2021	PEACEFM ONLINE	Air pollution second biggest cause of death in Africa’s booming cities.	Impact
20 October 2021	MYJOY ONLINE	US government and EPA commission air pollution quality monitoring station	Policy
11 June 2021	MYJOY ONLINE	Parliament moves to ascertain claims of dust pollution against new cement in Weija.	Policy
24 November 2021	MYJOY ONLINE	Accra is Africa’s 3rd most polluted city—2020 world air quality report.	Impact
